# GRANULOMATOUS PERIORAL DERMATITIS WITH EXTRA-FACIAL INVOLVEMENT IN CHILDHOOD: GOOD THERAPEUTIC RESPONSE WITH ORAL AZITHROMYCIN

**DOI:** 10.1590/1984-0462/;2018;36;4;00004

**Published:** 2018

**Authors:** Ana Carolina Xavier Milagre, Ana Paula Moura de Almeida, Hudson Dutra Rezende, Liana Moura de Almeida, Maria Auxiliadora Peixoto Peçanha

**Affiliations:** a Hospital Escola Álvaro Alvim, Campos dos Goytacazes, RJ, Brasil.

**Keywords:** Child, Perioral dermatitis, Azithromycin, Criança, Dermatite perioral, Azitromicina

## Abstract

**Objective:**

To present a case of granulomatous perioral dermatitis (GPD) with extra-facial involvement and good response to short-term treatment with oral macrolide.

**Case description:**

A 9-year-old girl presented with exuberant GPD with extra-facial involvement. During follow-up, she received multiple ineffective therapies, but showed significant improvement of the lesions after the use of azithromycin for five days.

**Comments:**

GPD is an inflammatory dermatological condition represented by papulo-erythematous eruptions on perioral, nasal and periorbital regions, more prevalent in children and adolescents. It rarely extends to the genital region, trunk, and extremities, which characterizes its extra-facial manifestation. Its etiology is unknown, but it seems to have a correlation with the use of topical corticosteroids and other agents.

## RESUMO

**Objetivo:**

Apresentar um caso de dermatite perioral granulomatosa (DPG) com acometimento extrafacial e resposta terapêutica satisfatória ao uso de macrolídeo oral por curto período.

**Descrição do caso:**

Escolar de nove anos, sexo feminino, com quadro exuberante de DPG com acometimento extrafacial. Durante o período de evolução, submeteu-se a múltiplas terapêuticas ineficazes, apresentando melhora significativa das lesões após o uso de azitromicina por cinco dias.

**Comentários:**

A DPG é uma afecção dermatológica inflamatória representada por erupções papuloeritematosas em região perioral, nasal e periorbitária, mais comum em crianças e adolescentes. Raramente estende-se à região genital, ao tronco e às extremidades, caracterizando o comprometimento extrafacial. De etiologia ainda desconhecida, parece apresentar correlação com uso de corticosteroides tópicos e outros agentes.

## INTRODUCTION

Granulomatous perioral dermatitis (GPD), a variant of classical perioral dermatitis, is a benign inflammatory disease affecting children in pre-pubertal age. Clinically, it is characterized by the presence of monomorphic, erythematous micro-papules, usually asymptomatic, which affect the central region of the face, especially the areas around oral, nasal and orbital cavities.^1^ ^,^ ^2^ Extra-facial involvement is rare, there are few cases reported in the literature with involvement of genital area, upper trunk, nape, and upper limbs.^1^ ^,^ ^3^ ^,^ ^4^

Its etiology remains controversial and its course is limited, but, since it causes important facial and esthetic impairment, in most cases treatment is advisable.

This study aims to describe a case of GPD with extra-facial manifestation which presented good therapeutic response to a short-term treatment with oral macrolide.

## CASE DESCRIPTION

**Figure 1:**
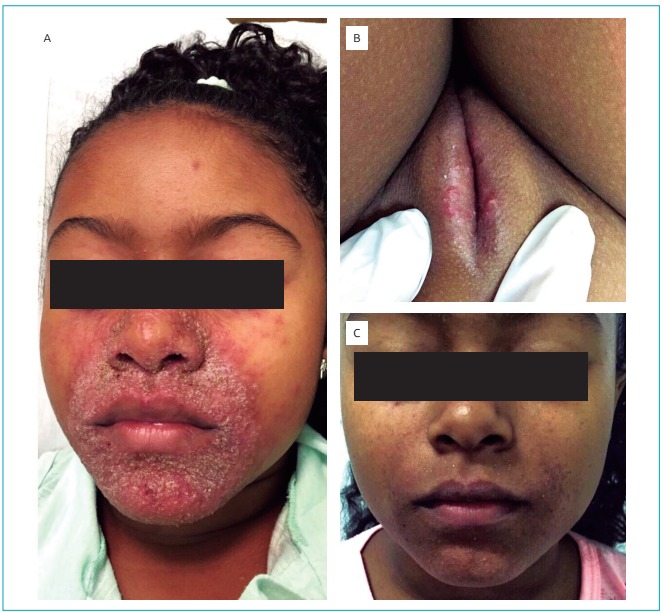
(A) Erythematous papular eruptions grouped in perioral, nasal and periorbital regions; (B) lesions in vulvar region; (C) patient after oral azithromycin for 5 days.

A 9-year-old female black-skinned patient presented with monomorphic, erythematous/desquamative papular eruptions grouped in perioral and periorbital regions for a year, with late progression onto the genital region (Figures 1A and 1B), not accompanied by any other symptoms.

Due to the exuberance of lesions, the patient was experiencing important social limitation, pictured by her distancing from groups of children’s recreation, parties and school environment. Over the disease course, multiple treatments were tried, including corticosteroids, imidazole and topical immunomodulators and systemic antibiotic therapy with cephalosporins, but lesions had no remission.

**Figure 2:**
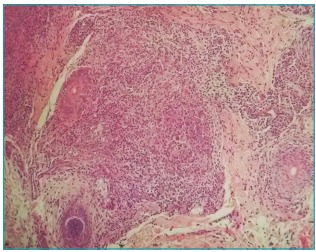
Lymphohistiocytic infiltrates around pilosebaceous follicles, constituting small granulomas. 107 x 84 mm (300 x 300 DPI).

Histopathological examination of a facial skin sample showed chronic and granulomatous findings. Dermal edema, vascular ectasia and lymphohistiocytic inflammatory infiltrates were noted around sebaceous follicles, configuring small granulomas surrounded by occasional neutrophils (Figure 2).

The initial presentation was devoid of symptoms, but the previous use of multiple topical agents caused local irritation, burn and pinching complaints. Topical tacrolimus 0.03% was prescribed under monotherapy, with significant improvement of erythema after one month. The appearance of new lesions in upper trunk and left upper limb in spite of the satisfactory facial response to therapy, led to the association of oral azithromycin, 320mg/day for five days, which finally provided disease remission (Figure 1C).

## DISCUSSION

First described in 1970 by Gianott,^5^upon finding granulomatous lesions in children with perioral dermatitis, GPD is synonymous with several names: Gianotti-type perioral dermatitis, Afro-Caribbean facial eruption, and childhood granulomatous periorificial dermatitis.^1^ ^,^ ^6^

An acneiform, micro-papular, monomorphic, erythematous eruption of reddish color, which may present yellow-brownish with fine surface scaling, is described in GPD. Lesions spread around the mouth and nose, rarely affecting other parts of the body such as neck, upper trunk, genital region or gain generalized distribution.^7^ It affects pre-pubertal children and most reports describe it in black patients.^8^ Most cases have spontaneous resolution and leave no scars.^9^

The etiology is still controversial, although some factors that would be related to the onset of symptoms have been proposed, such as infectious agents (*Candida* spp, demodex), fluoride toothpaste, chewing gums, amalgams, mercury, UVB radiation, and oral, topical and/or inhalational corticosteroids.^9^ ^,^ ^10^ The precipitating agent in our case is unknown, but the authors believed it to be a late worsening response due to the not proper use of topical corticosteroids.

Diagnosis is made clinically and there are no reports of systemic involvement.^9^ Histopathology is similar to that found in granulomatous rosacea type cases. Perivascular and perifollicular lymphohistiocytic infiltrates with vascular ectasia are seen. When granulomas are found, they are tuberculoid in shape, without central necrosis, identical to those seen in granulomatous rosacea, a rare variant of rosacea.^8^ ^,^ ^9^

Despite the histopathological similarity, cases of granulomatous rosacea differ from GPD because they progress to chronicity, affect middle-aged women, do not develop clinically with pustules and papules located in the lateral face, neck and submandibular regions, in addition to the possibility of telangiectasias. Another important differential factor compared to GPD is Lupus miliaris disseminatus faciei (LMDF). This, however, shows up in adolescents and young adults as papules symmetrically distributed across the face and caseum granuloma on histopathological examination, and healing with scar formation.^1^ GPD differential diagnoses should also consider: contact dermatitis, acne, seborrheic dermatitis and sarcoidosis.^11^

The initial treatment is removal of the causative agent, when it is identified. Topical antibiotics, mainly lotion or 0.75-1.00% metronidazole gel, have been the chosen treatment in most cases.^9^ The use of immunomodulators such as tacrolimus or topical pimecrolimus has also been suggested by some authors.^9^ ^,^ ^10^ In the patient herein presented, topical tracolimus was chosen due to extensive desquamation of the lesion.

Immunomodulatory agents are a more moisturizing vehicle compared to topical metronidazole, and because of its immunomodulatory activity it does not require corticosteroids. Extensive cases may require systemic antibiotic therapy, with tetracycline as well as second generation tetracyclines (minocycline, lymcycline and doxycycline associated with topical agents) being usually proposed. In cases of contraindication to tetracyclines, such as children younger than nine years old, due to teeth enamel discoloration and poor bone formation, the use of macrolides such as erythromycin and azithromycin has shown good results.^9^ The use of these antibiotics in such GPD cases is justified by their anti-inflammatory action, with changes in neutrophil chemotaxis and in the production of pro-inflammatory cytokines.^12^ For this purpose, azithromycin was the thrapy of choice because of the patient’s age and the medicine’s pharmacokinetics, which allows less frequent dosing and shorter therapy. In this case report, the remission of lesions occurred within a short period cycle.
