# ERRATA: Dermatite perioral granulomatosa com acometimento extrafacial na infância: boa resposta terapêutica com uso de azitromicina oral

**DOI:** 10.1590/1984-0462/2026/44/2017139-ERRATUM

**Published:** 2026-04-20

No artigo: Milagre ACX, Almeida APM, Rezende HD, Almeida LM, Peçanha MAP. Dermatite perioral granulomatosa com acometimento extrafacial na infância: boa resposta terapêutica com uso de azitromicina oral. Rev Paul Pediatr. 2018;36(4):511-514. https://doi.org/10.1590/1984-0462/;2018;36;4;00004, foi excluída a informação na Figura 1: "fotos A, B e C" a pedido da paciente, em razão da retirada de consentimento.

