# Supplementary material for: ERRATA: Dermatite perioral granulomatosa com acometimento extrafacial na infância: boa resposta terapêutica com uso de azitromicina oral
Source: Rev Paul Pediatr. 2026 Apr 20;44:e2017139-ER. [Article in Portuguese] doi: 10.1590/1984-0462/2026/44/2017139-ERRATUM (PMC13108584; doi:10.1590/1984-0462/2026/44/2017139-ERRATUM)
Supplement: Translation

# Translation: ERRATUM: Granulomatous perioral dermatitis with extra-facial involvement in childhood: good therapeutic response with oral azithromycin

**DOI:** 10.1590/1984-0462/2026/44/2017139-ER

In the manuscript: Milagre ACX, Almeida APM, Rezende HD, Almeida LM, Peçanha MAP. Granulomatous perioral dermatitis with extra-facial involvement in childhood: good therapeutic response with oral azithromycin. Rev Paul Pediatr. 2018;36(4):511-514. http://doi.org/10.1590/1984-0462/;2018;36;4;00004, the information in Figure 1, "photos A, B, and C" was removed at the patient's request due to the withdrawal of consent.
